# Supplementary material for: Structural basis of the T4 bacteriophage primosome assembly and primer synthesis
Source: Nat Commun. 2023 Jul 20;14:4396. doi: 10.1038/s41467-023-40106-2 (PMC10359460; doi:10.1038/s41467-023-40106-2)
Supplement: Supplementary file 1 — Supplementary Information [file 41467_2023_40106_MOESM1_ESM.pdf]

Supplementary Table 1. Cryo-EM data collection, refinement, and validation statistics for the ssDNA bound gp41 helicase complexes

	Close state gp41 hexamer with ssDNA (Step 3) (EMDB-27707) (PDB 8DTP)	Close state gp41 hexamer with ssDNA (Step 2) (EMDB-27708)	Open state gp41 hexamer with ssDNA (Step 3) (EMDB-27719) (PDB 8DUE)	Open state gp41 hexamer with ssDNA (Step 2) (EMDB-27720)
**Data collection and processing**				
Magnification	105,000	105,000	105,000	105,000
Voltage (kV)	300	300	300	300
Electron exposure (e^-/Å^2)	66	66	66	66
Defocus range (μm)	1.0 to 2.0	1.0 to 2.0	1.0 to 2.0	1.0 to 2.0
Pixel size (Å)	0.828	0.828	0.828	0.828
Symmetry imposed	C1	C1	C1	C1
Initial particle images (no.)	4,802,236	1,314,307	4,802,236	1,314,307
Final particle images (no.)	848,518	233,875	690,193	268,443
Map resolution (Å)	2.7	3.4	2.9	3.5
FSC threshold	0.143	0.143	0.143	0.143
Map resolution range (Å)	2.0-5.0	2.0-7.0	2.0-7.0	2.0-7.0
**Refinement**				
Initial model used (PDB code)	6QEM		6QEM	
Model resolution (Å)	2.9		3.1	
FSC threshold	0.5		0.5	
Model resolution range (Å)	2.0-5.0		2.0-7.0	
Map sharpening *B* factor (Å^2)	83.0		73.9	
Model composition				
Non-hydrogen atoms	20944		20902	
Protein residues	2592		2592	
Nucleotides	12		10	
Ligands	MG: 5 AGS:5		MG:5 AGS:5	
B factors (Å^2)				
Protein	57.2		80.9	
Nucleotide	95.8		69.1	
Ligand	52.8		49.4	
R.m.s. deviations				
Bond lengths (Å)	0.004		0.003	
Bond angles (°)	0.625		0.532	
Validation				
MolProbity score	2.06		2.42	
Clashscore	9.28		12.72	
Poor rotamers (%)	1.88		3.4	
Ramachandran plot				
Favored (%)	94.8		94.1	
Allowed (%)	5.2		5.9	
Disallowed (%)	0.0		0.0	

Supplementary Table 2. Cryo-EM data collection, refinement, and validation statistics for the gp41 helicase hexamer and the primosome in three states

	DNA-free gp41 hexamer (EMDB-27724) (PDB 8DUO)	Gp41-gp61-ssDNA (Pose 1) (EMDB-27737) (PDB 8DVF)	Gp41-gp61-ssDNA (Pose 2) (EMDB-27739) (PDB 8DVI)	Gp41-gp61-ssDNA (Pose 3) (EMDB-27751) (PDB 8DW6)
**Data collection and processing**				
Magnification	36,000	105,000		
Voltage (kV)	200	300		
Electron exposure (e^-/Å^2)	48	66		
Defocus range (μm)	1.4 to 2.4	1.0 to 2.0		
Pixel size (Å)	1.16	0.828		
Symmetry imposed	C1	C1	C1	C1
Initial particle images (no.)	71,335	4,802,236		
Final particle images (no.)	50,003	124,592	97,947	142,027
Map resolution (Å)	5.7	3.3	3.2	3.5
FSC threshold	0.143	0.143	0.143	0.143
Map resolution range (Å)	4.0-10.0	3.0-7.0	3.0-7.0	3.0-7.0
**Refinement**				
Initial model used (PDB code)	8DUE	6QEM, 6N9U, 2AU3	6QEM, 6N9U, 2AU3	6QEM, 6N9U, 2AU3
Model resolution (Å)				
FSC threshold 0.5	7.7	3.6	3.5	4.1
Model resolution range (Å)		3.0-7.0	3.0-7.0	3.0-7.0
Map sharpening *B* factor (Å^2)	210.0	99.4	90.7	109.0
Model composition				
Non-hydrogen atoms	20704	23693	23693	23693
Protein residues	2592	2914	2914	2914
Nucleotide	0	17	17	17
Ligands	MG: 5 AGS:5	MG:5 AGS:5 ZN:1	MG:5 AGS:5 ZN:1	MG:5 AGS:5 ZN:1
B factors (Å^2)				
Protein	257.1	81.5	81.5	81.5
Nucleotide	--	181.1	181.1	181.1
Ligand	205.6	52.7	52.7	52.7
R.m.s. deviations				
Bond lengths (Å)	0.004	0.004	0.004	0.004
Bond angles (°)	0.737	0.666	0.667	0.622
Validation				
MolProbity score	2.97	2.97	2.09	2.2
Clashscore	21.72	10.45	10.96	11.98
Poor rotamers (%)	7.1	1.78	1.78	3.2
Ramachandran plot				
Favored (%)	91.9	95.1	95.1	95.1
Allowed (%)	8.1	4.9	4.9	4.9
Disallowed (%)	0.0	0.0	0.0	0.0

Supplementary Table 3. Cryo-EM data collection, refinement, and validation statistics for the mutant primosome

	Close state mutated gp41 hexamer with ssDNA (EMDB-27707) (PDB 8G0Z)	gp61-ssDNA/RNA hybrids (EMDB-27756) (PDB 8DWJ)	Local refinement of the primase region in mutant T4 primosome (EMDB-29744)	Mutant primosome with E227Q helicase composite map (PDB 8GAO) (EMD-29902)
**Data collection and processing**				
Magnification	130,000	130,000	130,000	
Voltage (kV)	300	300	300	
Electron exposure ($e^-/Å^2$)	80	80	80	
Defocus range (μm)	1.0 to 2.0	1.0 to 2.0	1.0 to 2.0	
Pixel size (Å)	1.029	1.029	1.029	
Symmetry imposed	C1	C1	C1	
Initial particle images (no.)	5,706,491	5,706,491	5,706,491	
Final particle images (no.)	272,868	157,809	157,809	
Map resolution (Å)	3.6	3.9	4.1	
FSC threshold	0.143	0.143	0.143	
Map resolution range (Å)	3.0-7.0	3.0-8.0	3.0-8.0	
**Refinement**				
Initial model used (PDB code)	8DTP	6N9U, 2AU3	6N9U, 2AU3	
Model resolution (Å)	4.1	4.6	4.6	
FSC threshold	0.5	0.5	0.5	
Model resolution range (Å)	3.0-7.0	3.0-7.0	3.0-8.0	
Map sharpening *B* factor ($Å^2$)	121.6	126.3	70.5	
Model composition				
Non-hydrogen atoms	20944	3042		23987
Protein residues	2592	339		2931
Nucleotides	12	11		23
Ligands	MG:5 AGS:5	GTP:1 ZN:1		MG: 5 AGS:5 ZN: 1
B factors ($Å^2$)				
Protein	61.1	243.1		82.8
Nucleotide	101.5	275.8		186.7
Ligand	57.1	270.3		93.6
R.m.s. deviations				
Bond lengths (Å)	0.005	0.006		0.003
Bond angles (°)	0.721	1.268		0.592
Validation				
MolProbity score	2.22	2.07		2.35
Clashscore	12.05	14.2		12.60
Poor rotamers (%)	2.1	0.33		3.47
Ramachandran plot				
Favored (%)	94.4	93.77		94.55
Allowed (%)	5.6	5.64		5.45
Disallowed (%)	0.0	0.00		0.0

Supplementary Table 4. List of gp61 primase linker loop mutants.

Linker Loop		Residues	Mutagenic Primer Sequence
**WT**		^{106}PKELPK^{111}	
**1**	Shortened by 1 residue	PKE-PK	5′-ggtaaaagtcgtccaatagaaaaacctaaagaa-cctaaaca acccgagaagaaaataattaaatctc
**2**	Shortened by 4 residues	P----K	5′-gaaaagaaaaaggtaaaagtcgtccaatagaaaaacct-aa acaacccgagaagaaaataattaaatctcttccg
**3**	Extended by 2 residues	PKEGSLPK	5′-ggtaaaagtcgtccaatagaaaaacctaaagaaggttcccttcc taaacaacccgagaagaaaataattaaatctc
**4**	Extended by 5 flexible residues	PKEGGGGSLPK	5′-ggtaaaagtcgtccaatagaaaaacctaaagaaggtggcgga gggtcccttcctaaacaacccgagaagaaaataattaaatctc
**5**	Extended by 4 rigid residues	PKELPKELPK	5′-ggtaaaagtcgtccaatagaaaaacctaaagaacttcccaag gagctacctaaacaacccgagaagaaaataattaaatctc

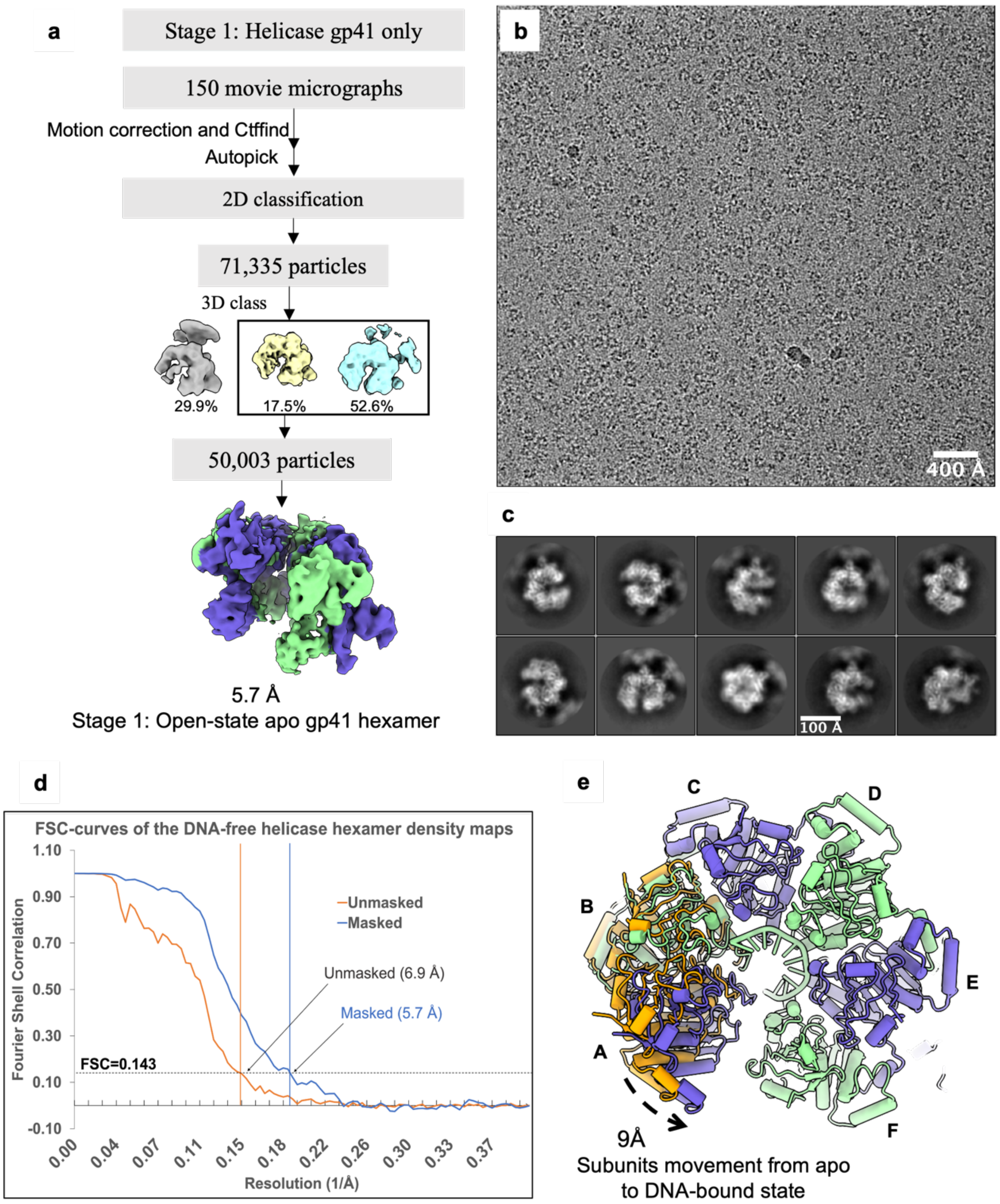

Supplementary Figure 1. Processing of EM images of the ATPγS-bound gp41 helicase sample in the absence of ssDNA. a) Data processing flowchart. **b**) A typical raw micrograph. **c**) Selected 2D class averages. **d**) Gold-standard Fourier shell correlation curve indicating an averaged resolution of 5.7 Å at the 0.143 correlation threshold. **e**) Comparison of the open spiral gp41 helicase before and after binding ssDNA; the two structural models are aligned based on subunit F. Subunits A-F are colored alternatively in blue or green. Subunits A and B in the gp41 helicase structure without ssDNA are shown in orange to better show the movement of these subunits when binding to ssDNA.

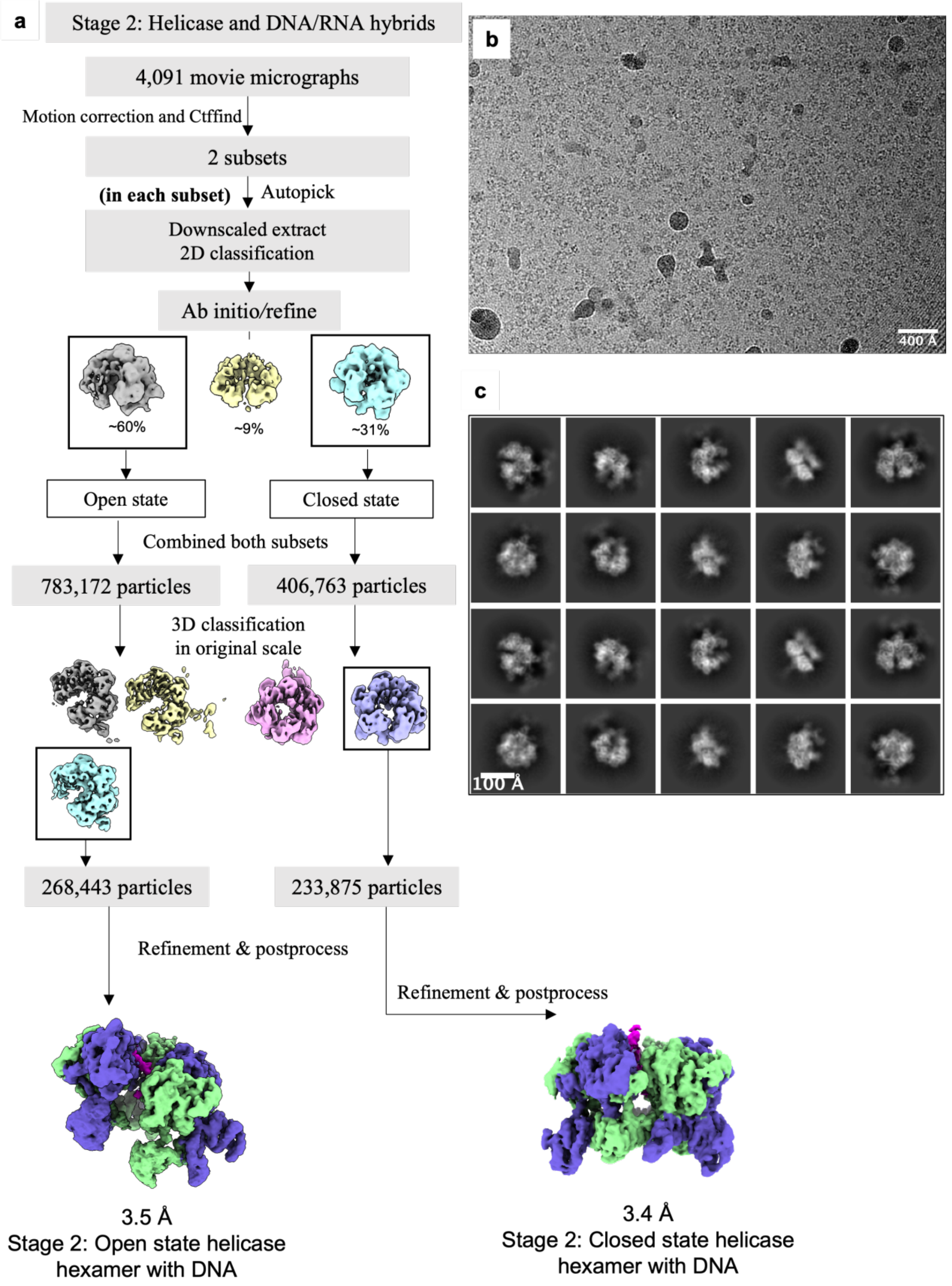

Supplementary Figure 2. Processing of cryo-EM images from the sample of the gp41 helicase mixed with the ssDNA/RNA primer. a) Data processing flowchart. **b)** A typical raw micrograph. A total of 4,091 such raw micrographs were recorded. **c)** Selected 2D class averages.

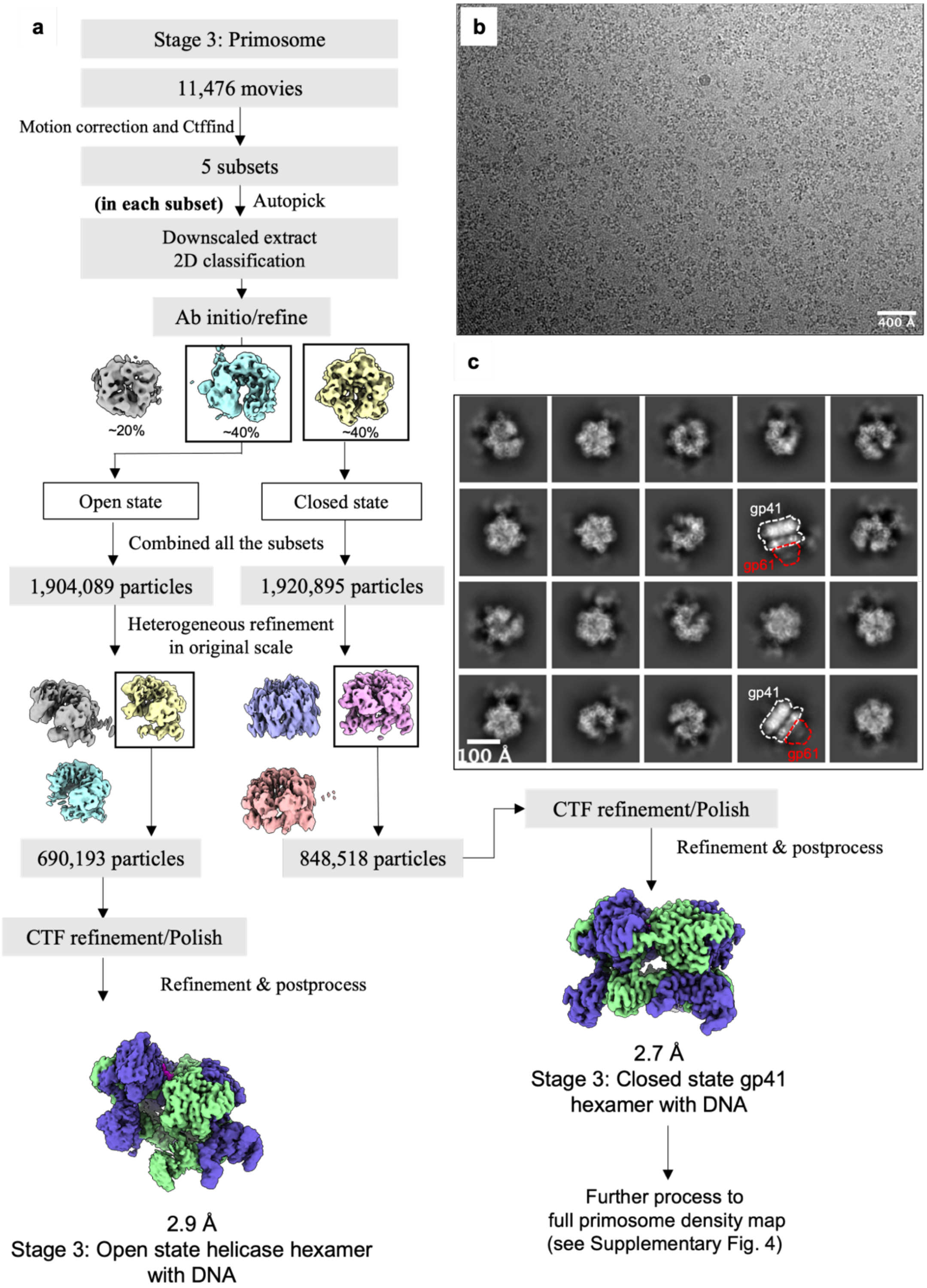

Supplementary Figure 3. Initial processing of cryo-EM images from the primosome assembly sample. a) Data processing flowchart for reconstructing the gp41 helicase within the primosome particles. **b**) A typical raw micrograph. A total of 11,476 such raw micrographs were recorded. **c**) Selected 2D class averages showing the presence of the gp61 primase in some averages.

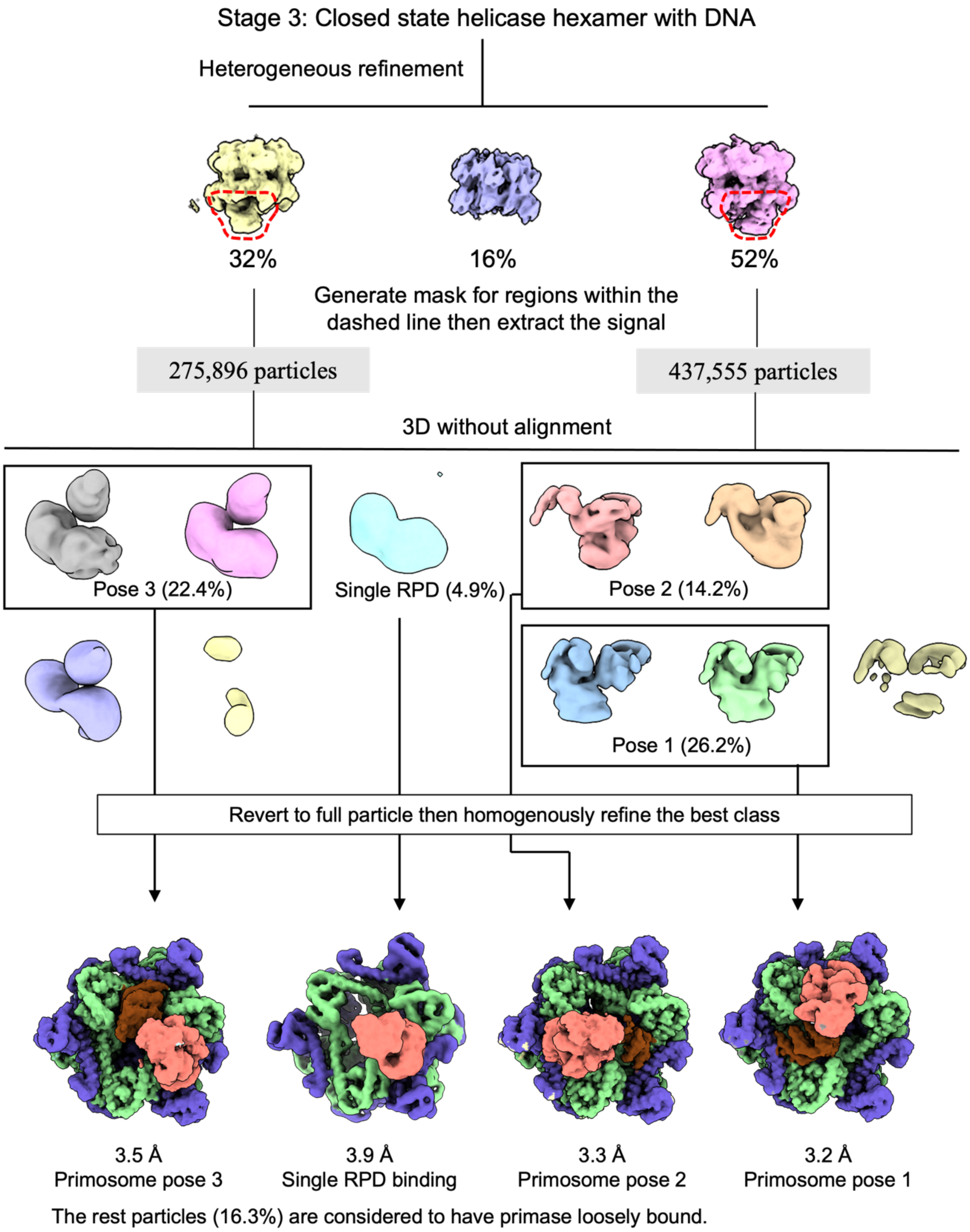

Supplementary Figure 4. Additional processing of cryo-EM images from the primosome assembly sample. Further processing of the closed-state particles within the primosome sample led to the identification of three major bipartite binding poses and one minor single-domain (RPD) binding pose of the gp61 primase within the T4 primosome.

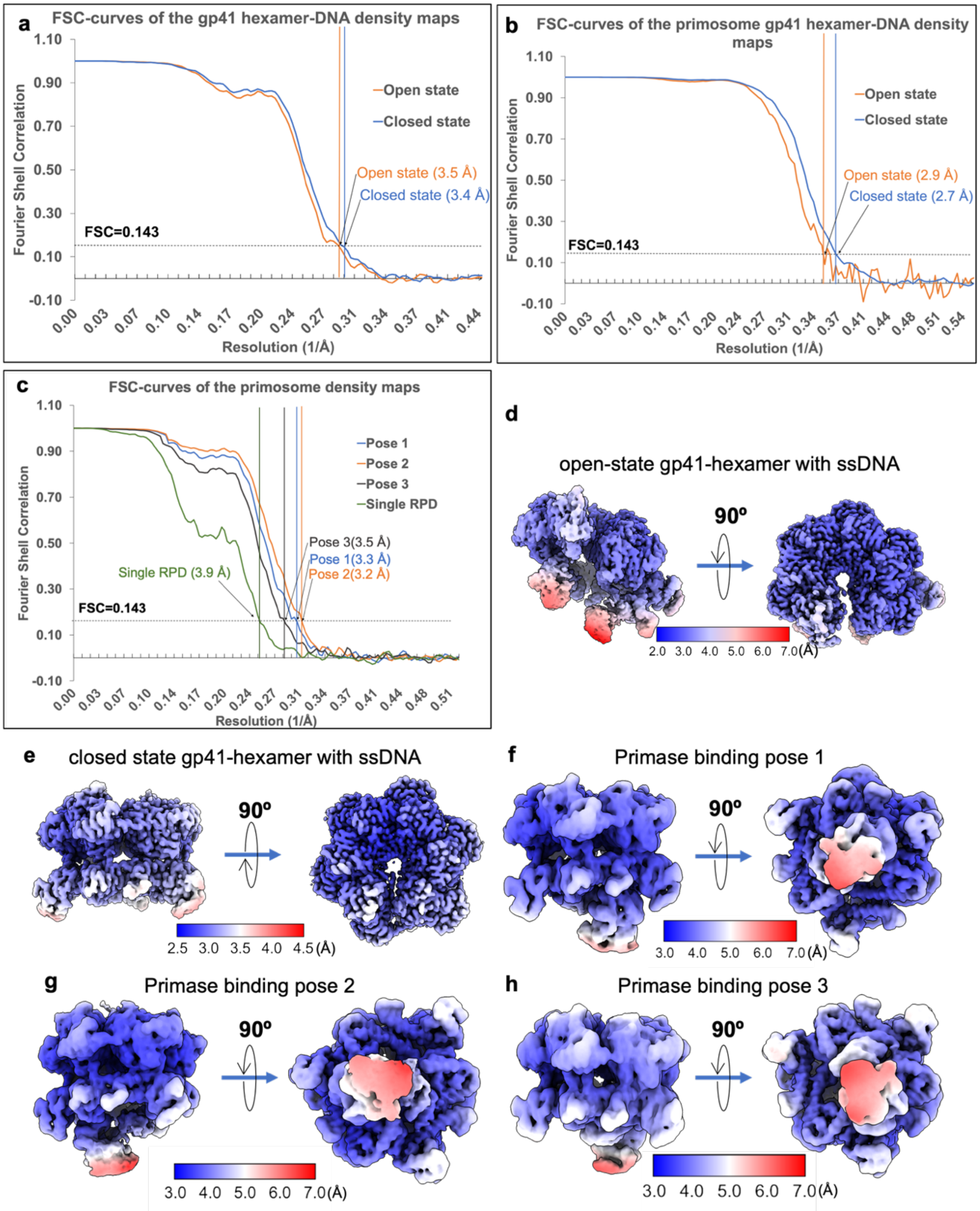

Supplementary Figure 5. Resolution estimation of the EM maps of the gp41 helicase-ssDNA complex and the primosome. FSC curves of the open- and closed-state gp41 helicase/ssDNA in **a)** the gp41/ssDNA sample and **b)** the primosome sample. **c)** FSC curves of the four primase-bound poses in the primosome sample. Local resolution maps of the primosome assembly intermediates: **d)** the open-state and **e)** the closed-state of the gp41 helicase/ssDNA sample; and the primase-bound **f)** pose 1, **g)** pose 2, and **h)** pose 3 in the primosome sample.

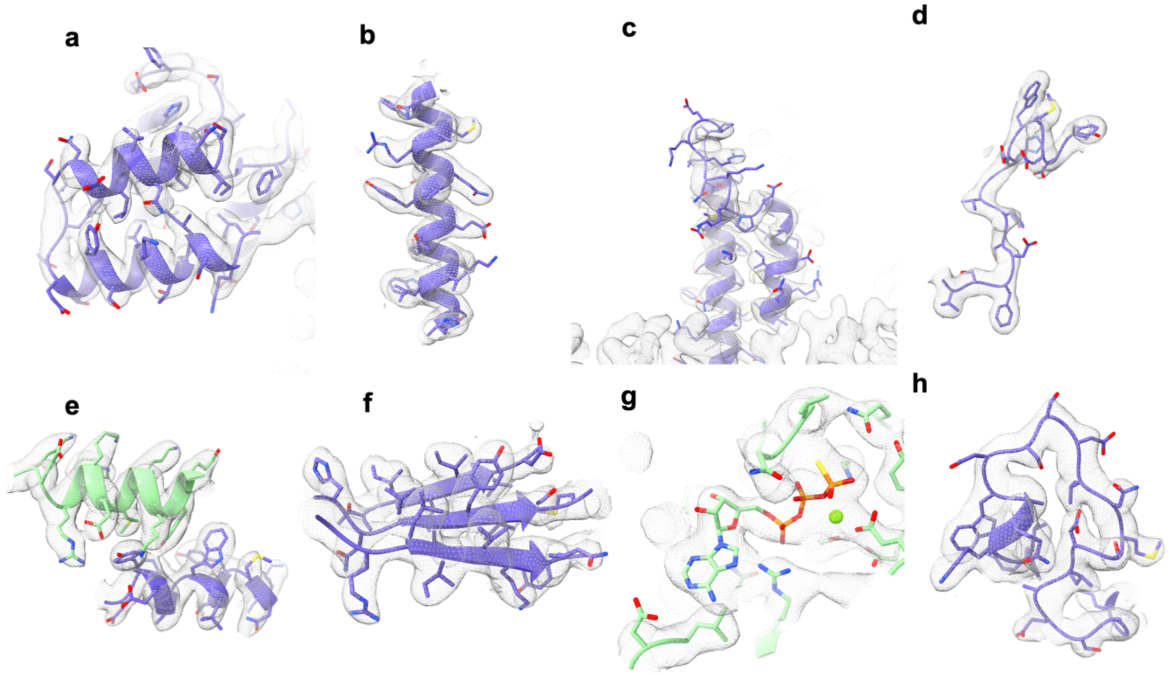

Supplementary Figure 6. Fitting of the gp41 helicase atomic model with selected regions of the EM map. Comparison of the EM map rendered at a threshold of 2σ (semitransparent grey surface) with selected regions of the gp41 helicase atomic model: **a)** N-terminal globular subdomain; **b-c)** two regions from the N-terminal helical subdomain; **d)** the loop linking the NTD and the linking helix; **e)** the linking helix and the interacting helix of the adjacent subunit; **f)** the β-sheets within the RecA-like CTD; **g)** an ATPγS molecule and its binding pocket; and **h)** the DNA interacting loop 1. All density regions are extracted from the closed-ring helicase structure in EM map 3-II.

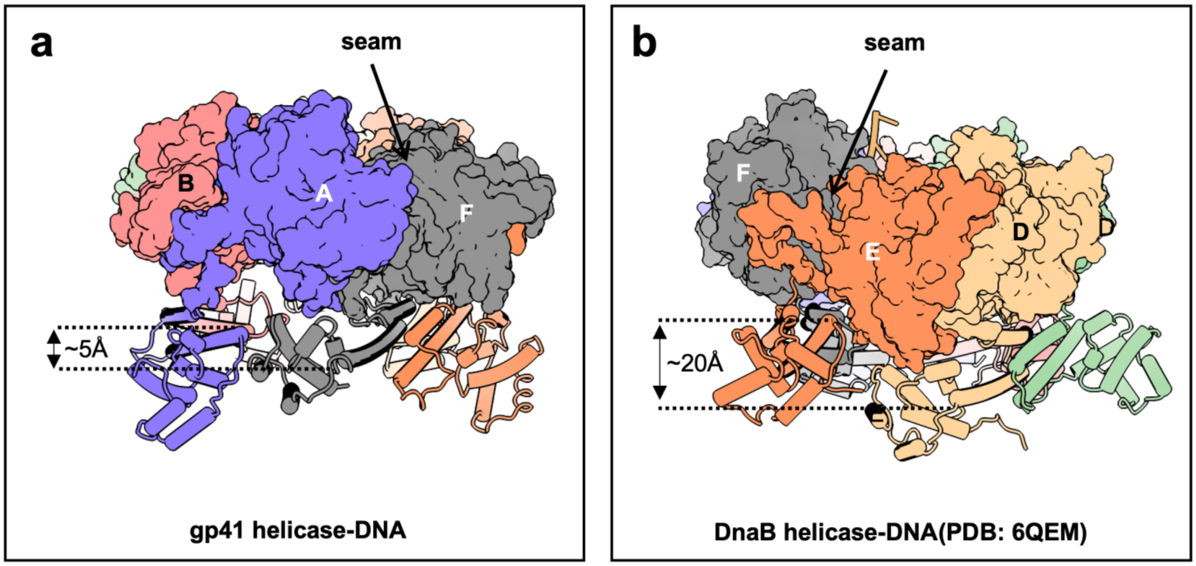

Supplementary Figure 7. Comparison of the T4 gp41 and *E. coli* DnaB helicases in translocation-competent active states. Side views showing **a)** an almost planar ring arrangement of the gp41 helicase with a small (5 Å) lateral staggering (this study) and **b)** a twisted non-planar ring of the DnaB helicase with a large (20 Å) lateral staggering (PDB entry 6QEM) between the adjacent subunits. Both the gp41 and DnaB helicases assemble as trimer-of-dimers in their active state. The three dimers consist of subunit A-B, subunit C-D and subunit E-F. The seam identified as the nucleotide-free interface is between subunits A and F, which belong to two separate dimers, in the gp41 helicase and between subunits E and F, which belong to a single dimer, in the DnaB helicase.

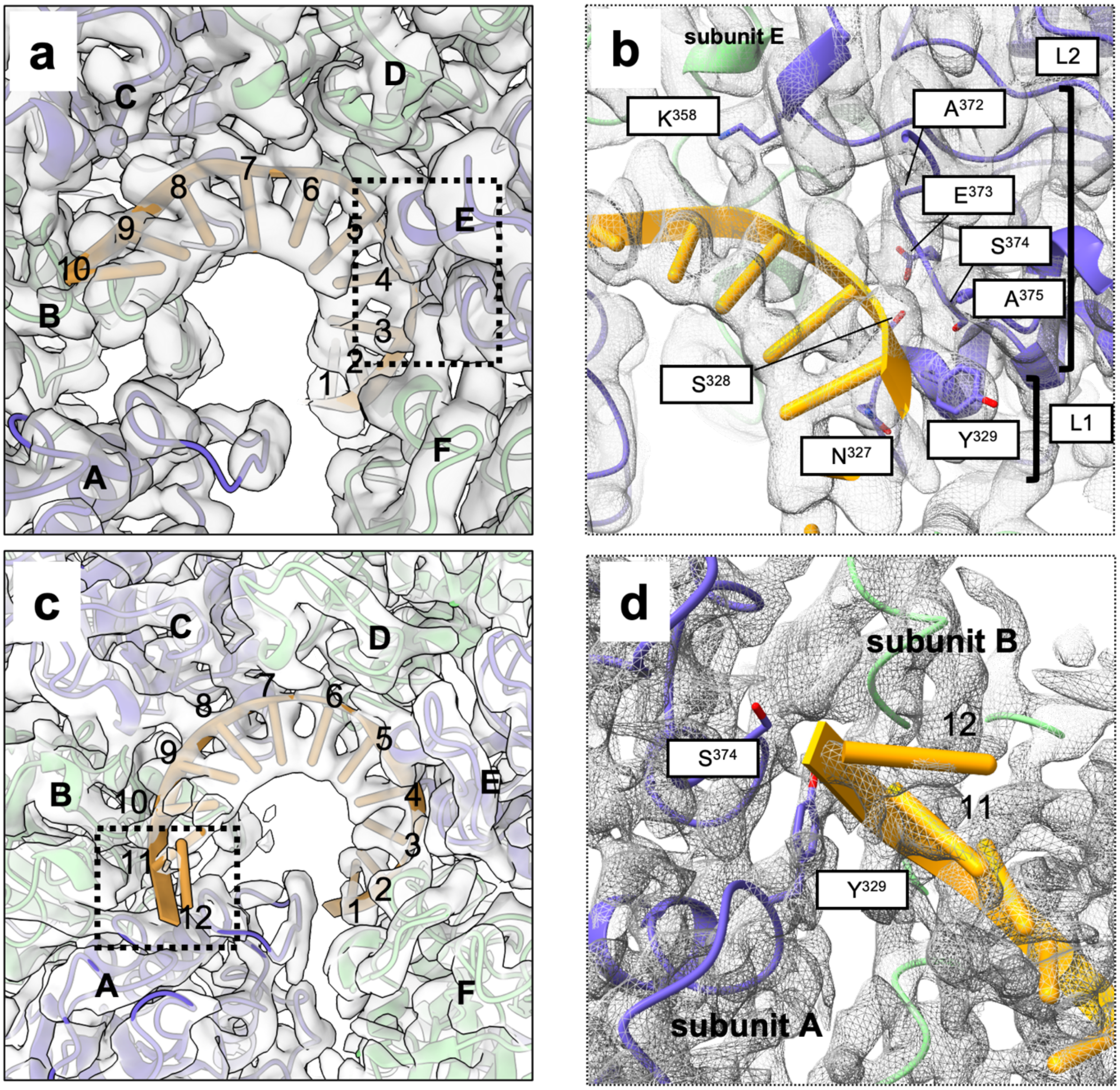

Supplementary Figure 8. The ssDNA binding mode in the open and closed states of the gp41 helicase. a) An overall view and **b)** a zoomed-in view of the ssDNA binding region in the central channel of the open spiral gp41 helicase structure. **c)** An overall view and **d)** a zoomed-in view of the ssDNA binding region in the central chamber of the closed-ring gp41 helicase structure. The EM maps are shown as transparent grey surface in (a) and (c) and as grey meshes in (b) and (d); the atomic models of DNA are sticks; and the residues interacting with the DNA are sticks with the residue names labeled in one letter codes. L1 and L2 refer to the DNA-translocating loops 1 and 2, respectively.

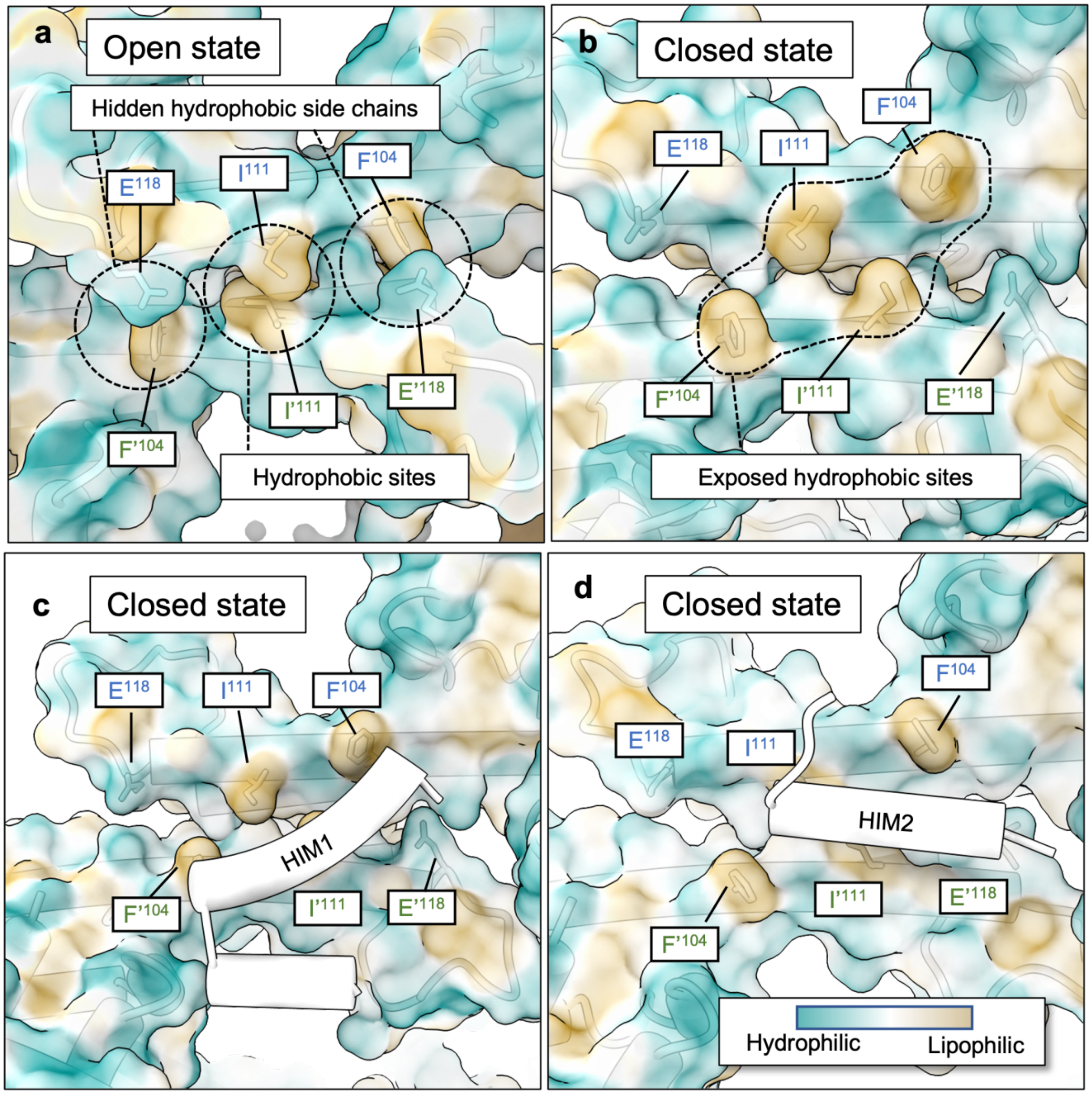

Supplementary Figure 9. Surface hydrophobicity of the gp41 NTD dimers in various states. a) In the open spiral, the helical hairpins in one NTD dimer are in an "X" configuration where the two Ile111 residues from each subunit face each other, and the two hydrophobic Phe104 residues are shielded by the two hydrophilic Glu118 residues from each subunit. **b)** In the closed-ring gp41 helicase, the helical hairpins are parallel exposing the four hydrophobic Ile111 and Phe104 residues creating the binding site for **c)** the helicase-interacting motif HIM1 and **d)** the HIM2 of the gp61 primase. Interaction surfaces of HIM1 and HIM2 are colored based on the level of the hydrophobicity from dark cyan to wheat (inset).

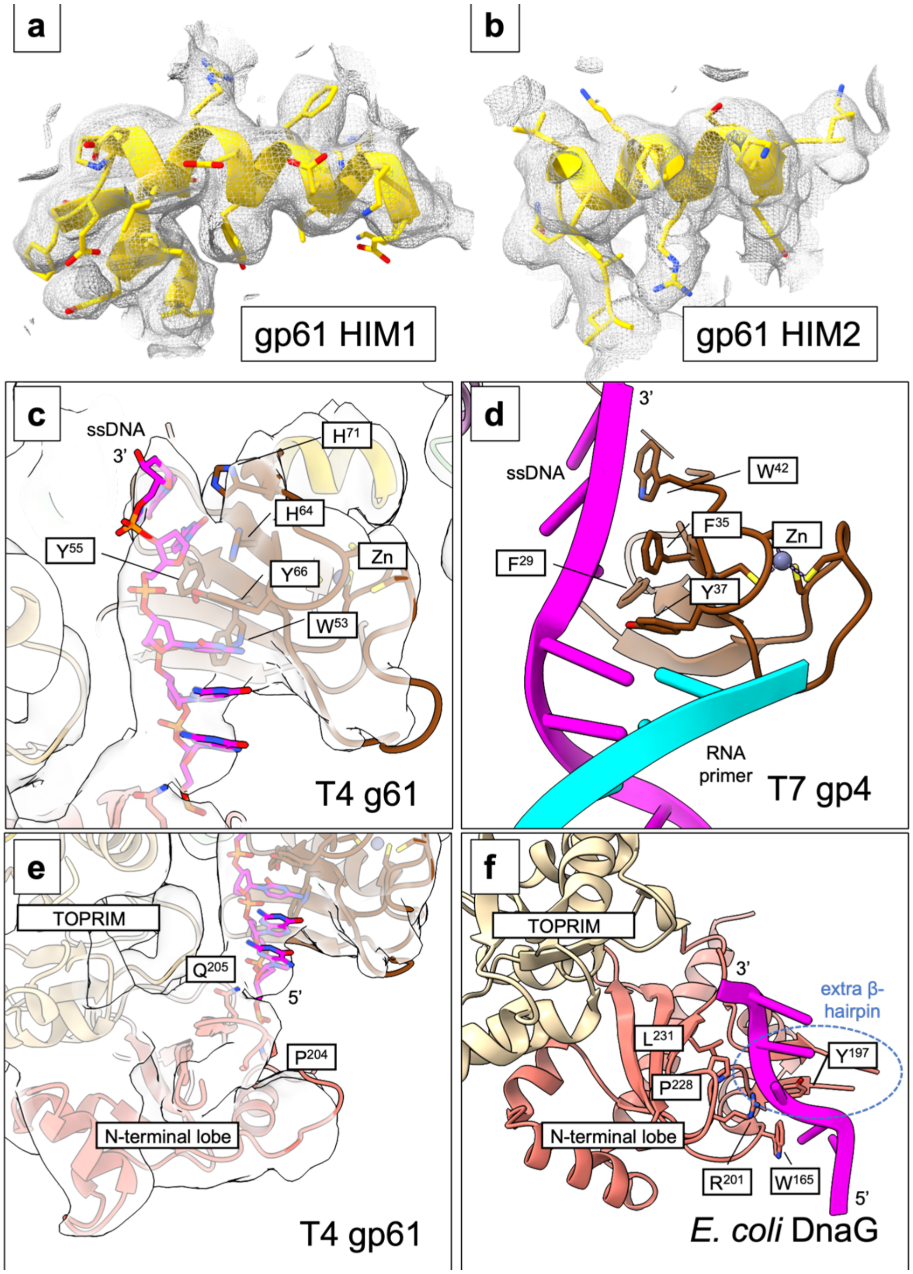

Supplementary Figure 10. Local EM densities of the gp61 primase. EM densities of **a)** gp61 HIM1 and **b)** gp61 HIM2. Comparison of the interactions between the conserved ZBD and the ssDNA in **c)** the gp61 primase (this study, primosome pose 1) and **d)** in the T7 gp4 (PDB 6N9U). The ssDNA interacts with the conserved residues Tyr66 in the gp61 primase and Tyr37 in the gp4 primase. Comparison of the interaction between the RPD and the ssDNA in **e)** the gp61 primase (this study, primosome pose 1) and **f)** the *E. coli* DnaG primase (PDB 3B39). In both structures, the ssDNA interacts with the β-hairpin in the RPD N-terminal lobe. We note that the DnaG primase has an additional β-hairpin.

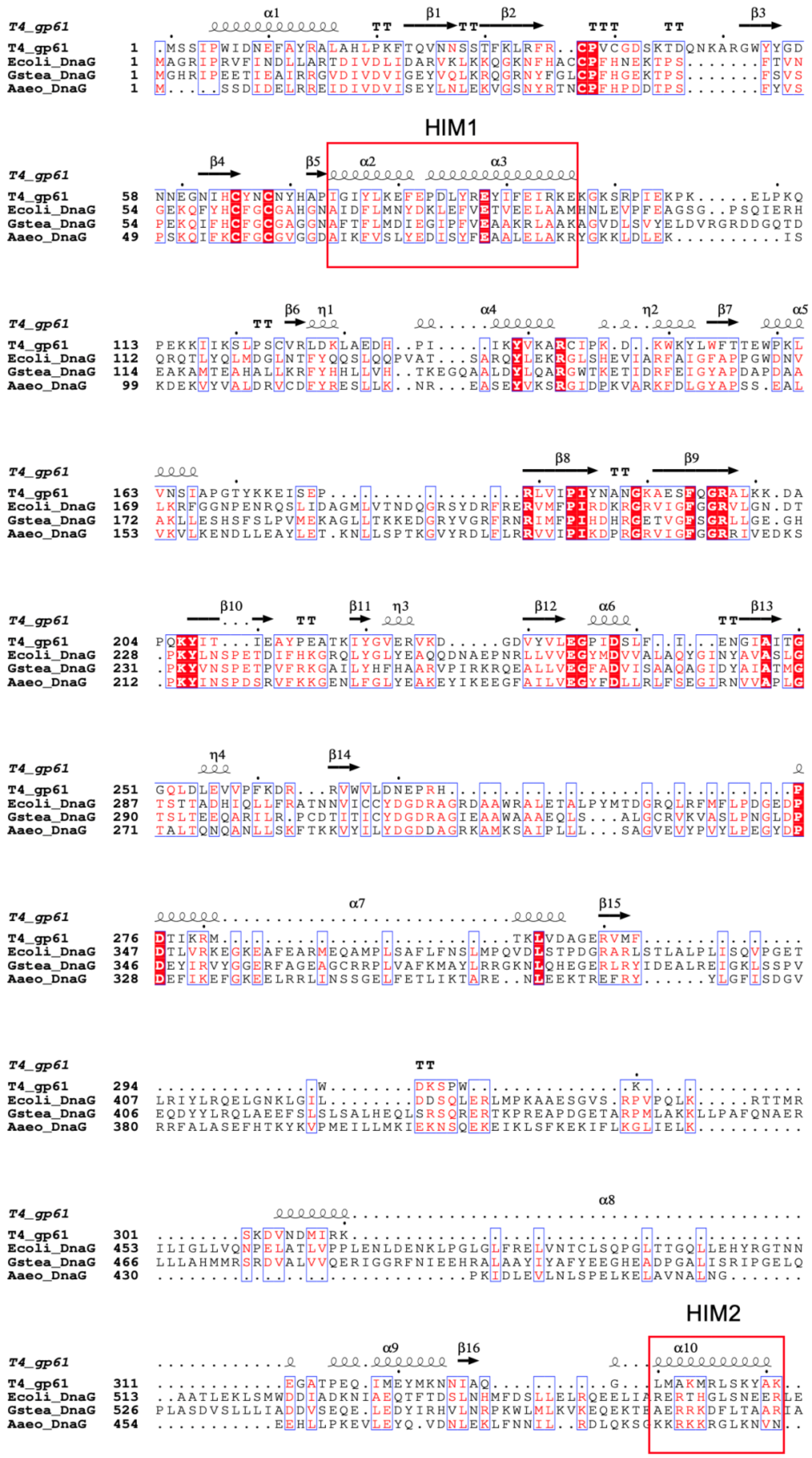

Supplementary Figure 11. Sequence alignment of the T4 gp61 with several bacterial DnaG primases. The helix-interacting motifs HIM1 and HIM2 of gp61 are highlighted by red boxes in the sequence alignment demonstrating that the interfacial residues of Pro83, Arg87, Ile90, Phe91, and Arg94 are not conserved in the DnaG primases. Ecoli: *Escherichia coli*; Gstea: *Geobacillus stearothermophilus*; Aaeo: *Aquifex aeolicus*. Note that the gp61 HIM2 structurally aligns with the C-terminal HID of the DnaG primases, but their sequences are not conserved.

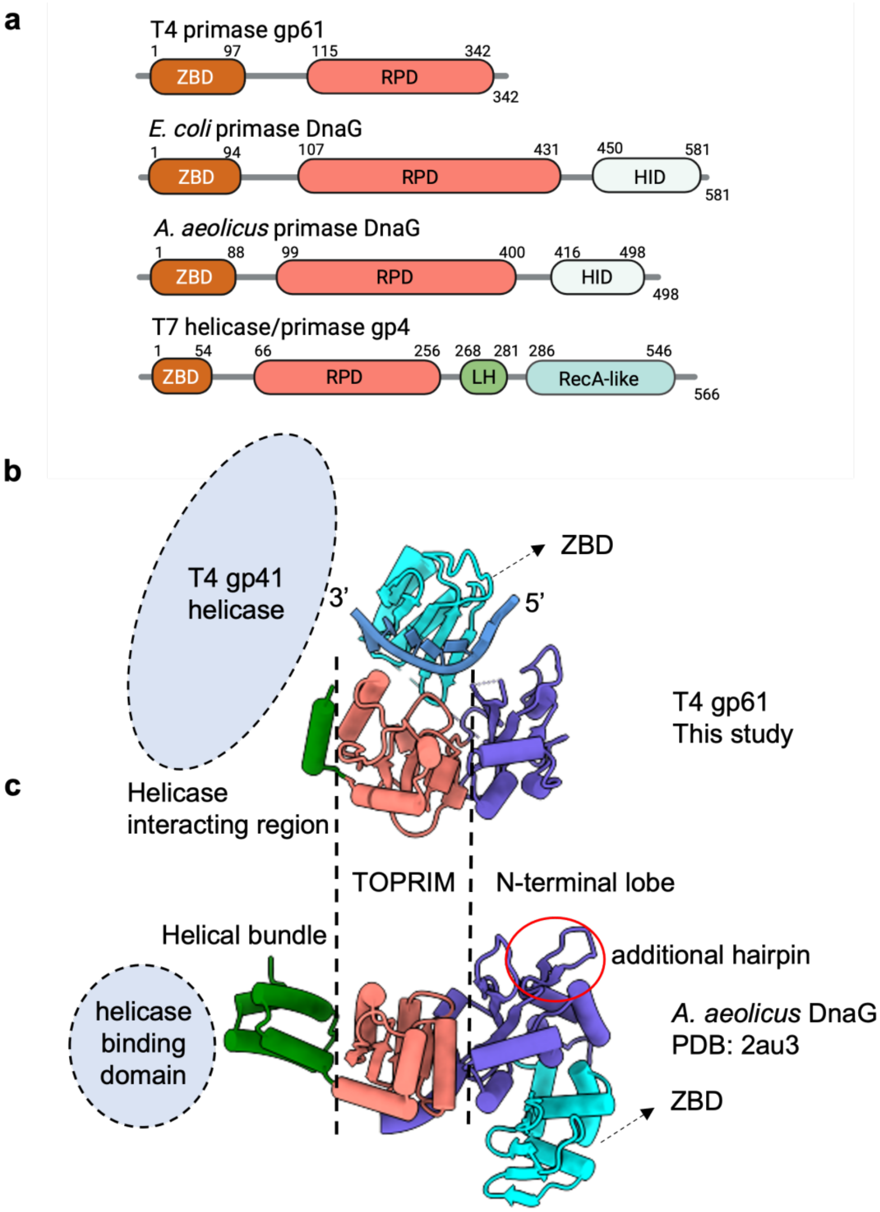

Supplementary Figure 12. Structural comparison of the T4 gp61 and the bacterial DnaG primases. a) Domain architecture of the T4 gp61, T7 gp4, and two bacterial DnaG primases. The structures of **b)** the gp61 primase (this study) and **c)** the DNA-free *A. aeolicus* DnaG primase (PDB 2AU3) are aligned and shown separately for clarity. The TORPIM domain is similar in both structures, and the DnaG helicase interaction domain (HID) functions similarly to the gp61 HIM2. The N-terminal lobe of the DnaG RPD contains an ssDNA-binding β-hairpin (red circle) that is absent in the gp61 primase. The location of the ZBD is different between the primases; the ssDNA-binding surfaces of the gp61 RPD and ZBD face the ssDNA.

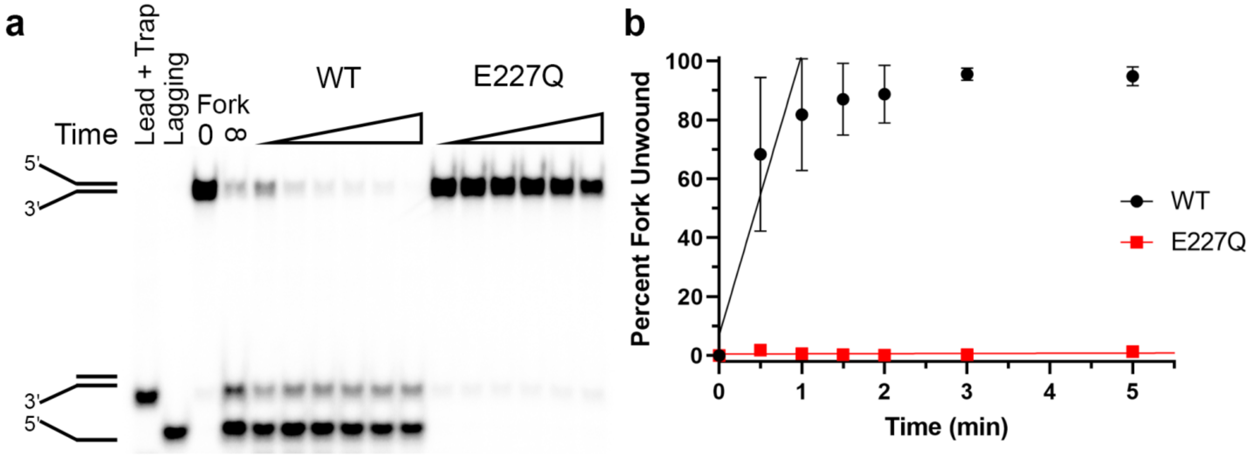

Supplementary Figure 13. Unwinding activity of the gp41(E227Q) helicase mutant. a) Representative native polyacrylamide gel analysis of the unwinding activity of WT or gp41(E227Q) helicase at time 30, 60, 90, 120, 180, and 300 s. Standards: Lane 1 – 50 nM fork lead and 500 nM trap; Lane 2 – 50 nM fork lag; Lane 3 – 50 nM fork DNA and 500 nM trap; Lane 4 – 50 nM fork DNA and 500 nM trap heated at 95 °C for 10 min. This experiment was repeated three times. **b)** The percent of fork DNA unwound calculated from quantified native gels in triplicate graphed versus time demonstrates that the gp41(E227Q) helicase mutant has no unwinding activity compared to the WT gp41 helicase. Source data are provided as a Source Data file.

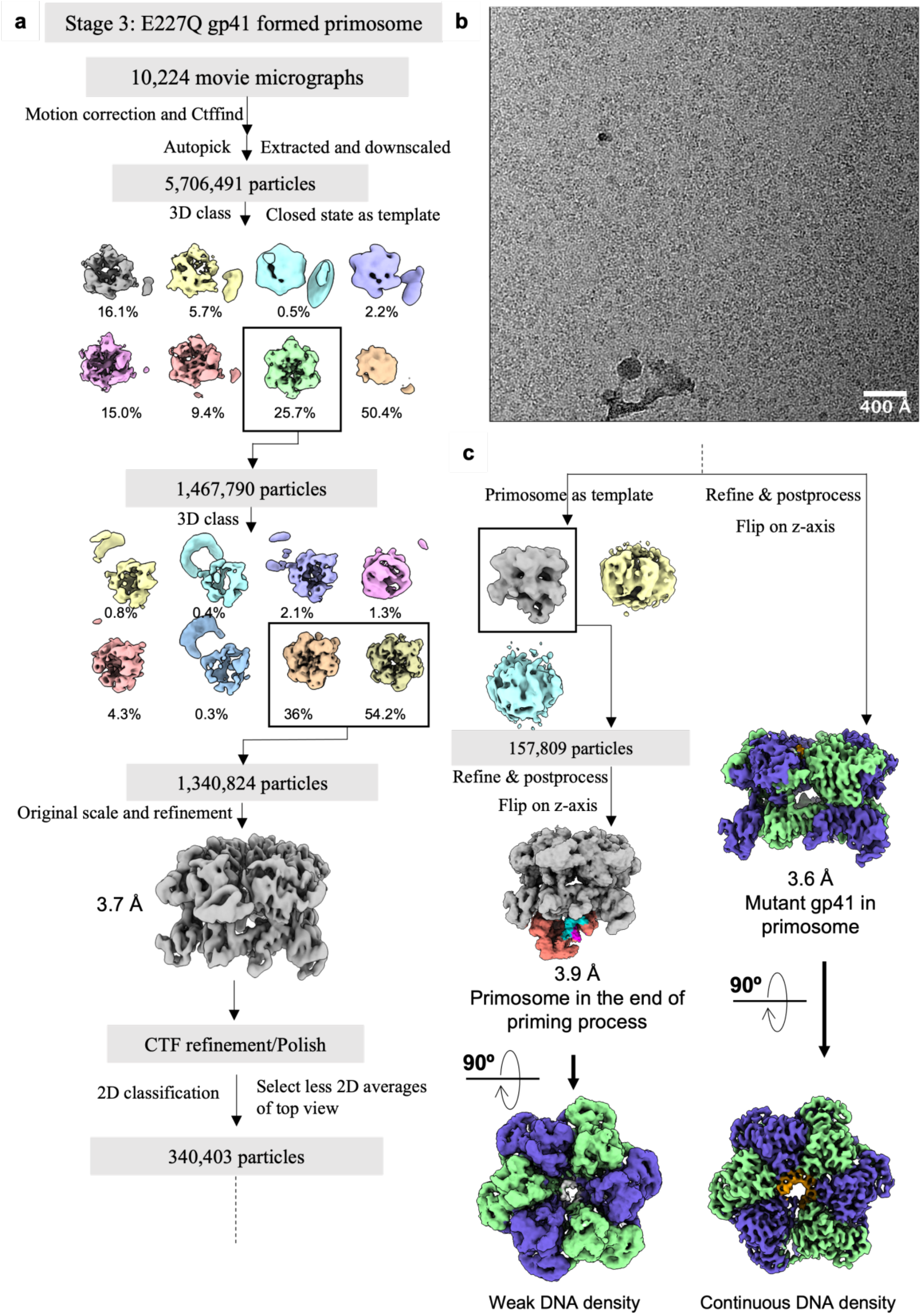

Supplementary Figure 14. Processing of the cryo-EM images from the mutant T4 primosome assembly sample. a) Data processing flowchart. **b)** A typical raw micrograph. A total of 10,224 such micrographs were recorded. **c)** Additional data processing steps leading to the EM maps of the gp41 helicase(E227Q) alone (right) and the mutant primosome consisting of the gp41 helicase(E227Q) and the gp61 primase (left).

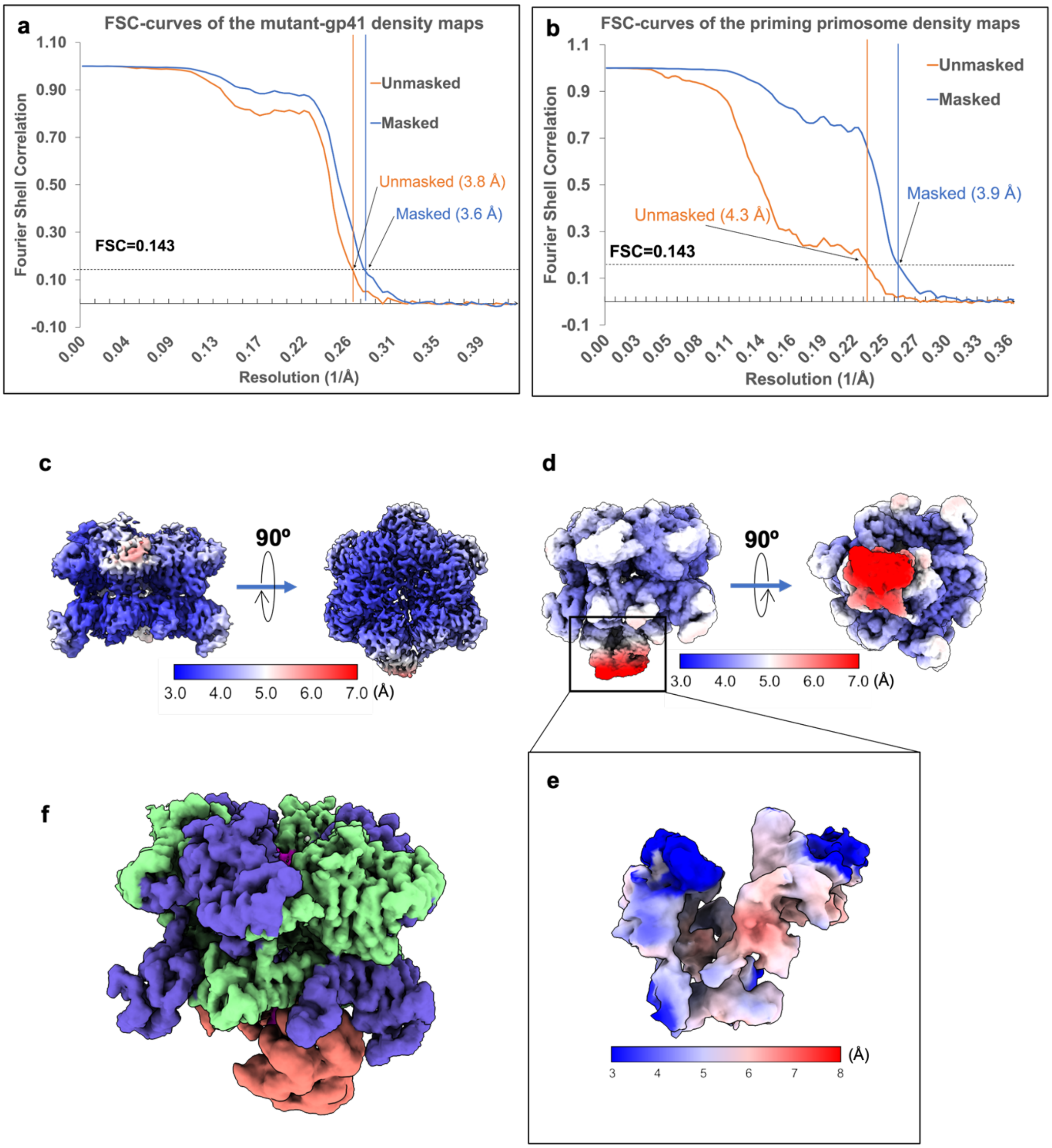

Supplementary Figure 15. Resolution estimation of the mutant T4 primosome EM maps. FSC curves of the 3D EM maps of **a)** the ssDNA-bound gp41 helicase(E227Q) and **b)** the mutant primosome. The mutant primosome consists of a well-defined primase region and averaged helicase region, indicating multiple binding orientations of the primase. Local resolution maps of **c)** the ssDNA-bound gp41 helicase(E227Q), **d)** the mutant primosome, and **e)** the focus refined primase region. **f)** The focus refined primase and helicase were combined into a composite map and colored by chain.

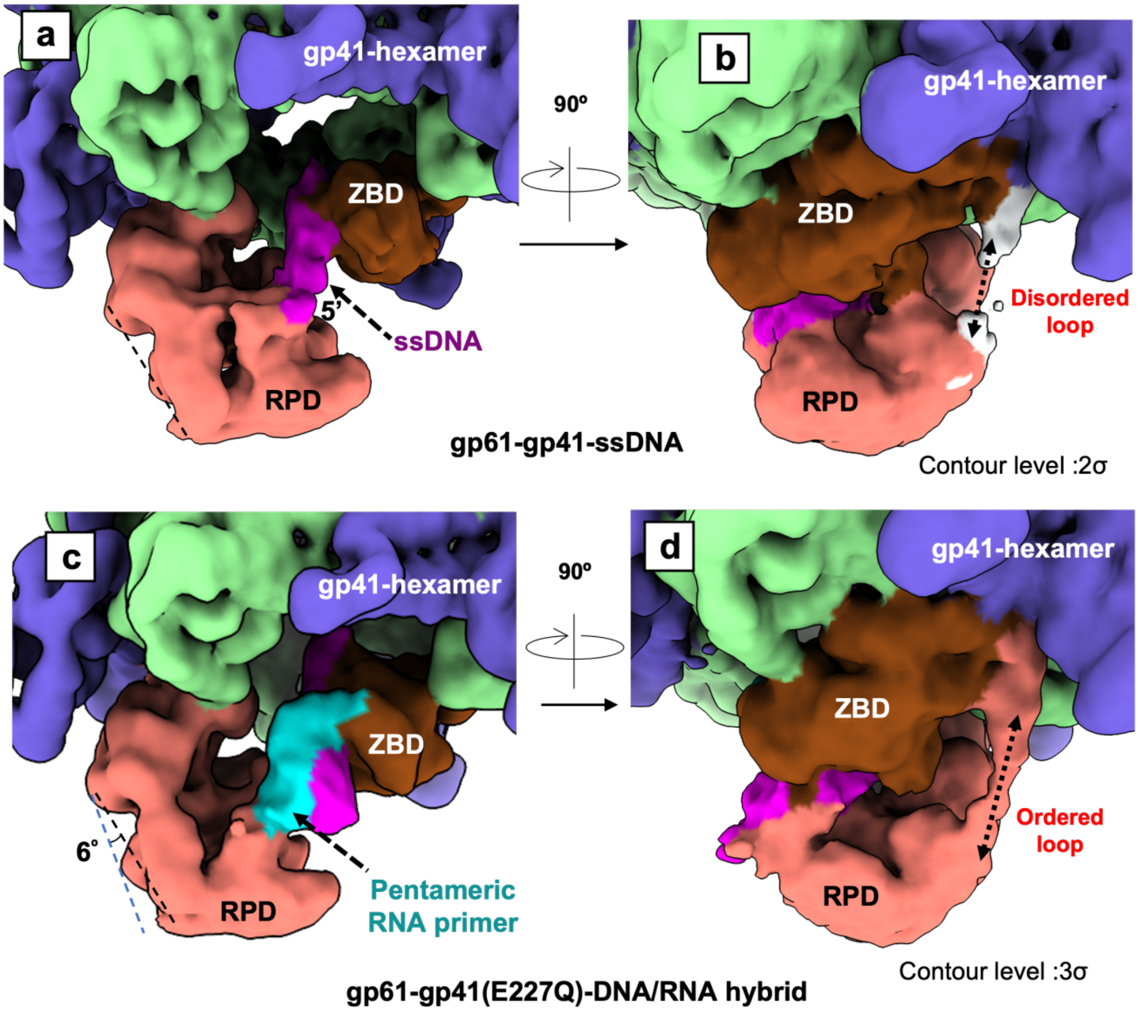

Supplementary Figure 16. Comparison of the EM maps of the T4 primosome in potential DNA-scanning and post RNA primer-synthesis modes. a-b) Two views of the EM map of the WT T4 primosome likely in a DNA-scanning mode. **c-d)** Two views of the EM map of the mutant T4 primosome likely in a post RNA primer-synthesis mode. Note that the gp61 linker loop connecting the ZBD and RPD is disordered in **b)** the potential DNA-scanning mode, but becomes well-ordered in **d),** likely a post RNA primer-synthesis mode.

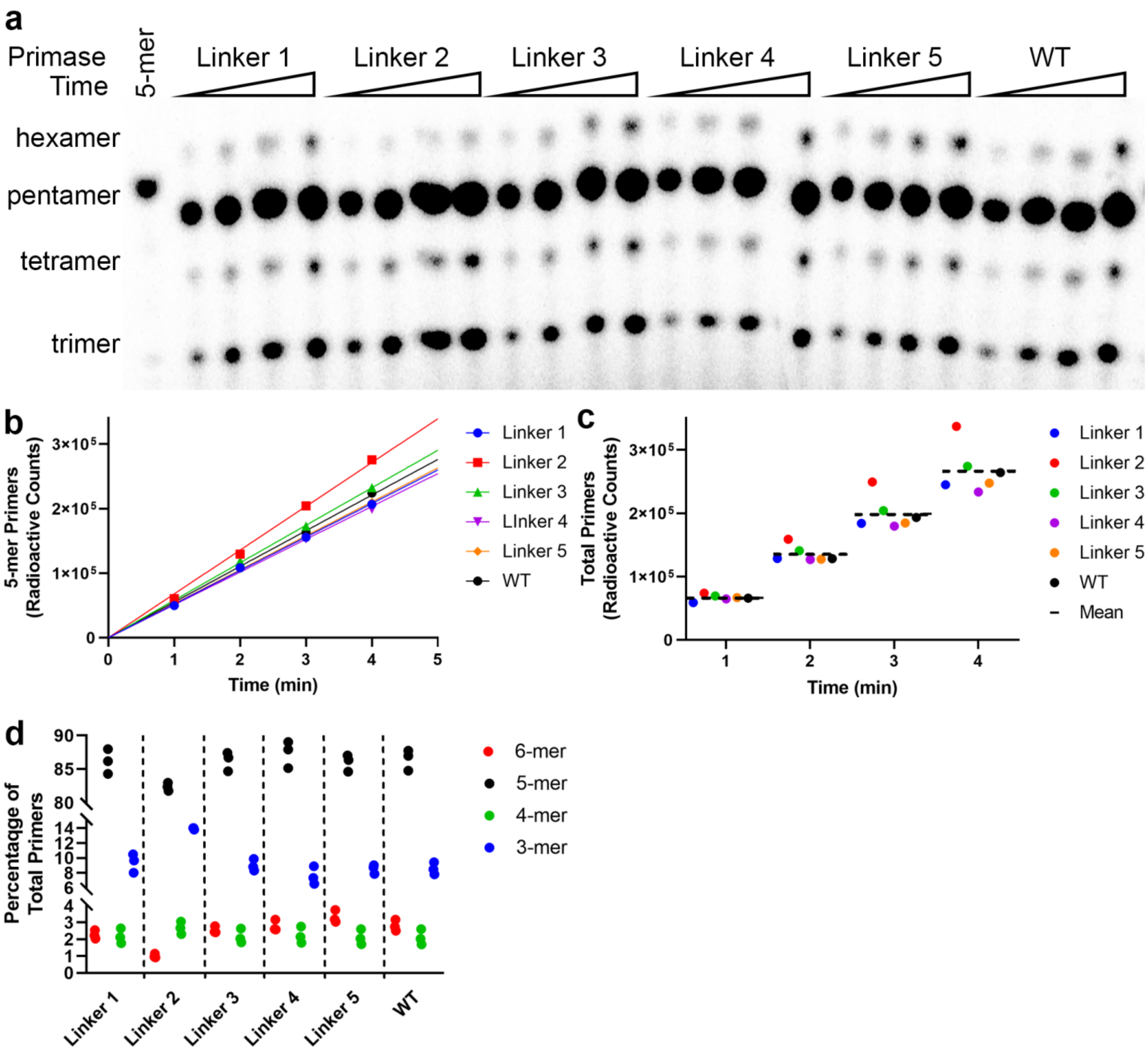

Supplementary Figure 17. Priming activity of the series of gp61 primase linker loop mutants. a) Representative denaturing polyacrylamide gel analysis of the priming activity of the series of gp61 primase linker loop mutants at time 1-, 2-, 3-, and 4-min. Standard: Lane 1 – radiolabeled synthetic RNA 5-mer oligo. This experiment was repeated three times. **b)** Graph of the pentamer primers synthesized by WT and each gp61 primase linker loop mutant versus time demonstrates that the reaction is linear over the 4 min assay time. **c)** Dot plot of the total primers synthesized by WT and each gp61 primase linker loop mutant versus time demonstrates that the overall priming activity of the primase mutants was not affected by the changes made to shorten or extend the linker loops. **d)** Dot plot compiled from triplicate priming assays depicting the percentage of various length primers synthesized by the indicated WT and linker loop mutant primases after 4 min. Primase mutant linker 2, where the linker loop was shortened by four residues, synthesized slightly less pentamer and hexamer primers and more trimer primers with respect to the WT and other mutant primases. Source data are provided as a Source Data file.
