# Supplementary material for: Structural basis of the T4 bacteriophage primosome assembly and primer synthesis
Source: Nat Commun. 2023 Jul 20;14:4396. doi: 10.1038/s41467-023-40106-2 (PMC10359460; doi:10.1038/s41467-023-40106-2)
Supplement: Supplementary file 3 — Description of Additional Supplementary Files [file 41467_2023_40106_MOESM3_ESM.pdf]

Description of Additional Supplementary Files

File Name: Supplementary Movie 1
Description: **The assembly process of the T4 primosome.** The movie starts with the inactive open-spiral gp41 helicase, morphs to the ssDNA template bound and still inactive helicase, then transitions to the active closed-ring helicase with a large conformational change, and finally shows the gp61 primase binding to the active helicase hexamer; this completes the assembly of the T4 primosome.

File Name: Supplementary Movie 2
Description: **The two functional states of the T4 primosome.** The movie begins by showing the morph among the three binding poses of the gp61 primase on the gp41 helicase hexamer in the primosome state 1, which is suggested to be a DNA-scanning mode. Then, the scene transitions to focus on the primase binding region, morphing between states 1 and 2. State 2 is likely a post RNA primer-synthesis state. Transition from state 1 to 2 is repeated twice to highlight the rotation of the RPD primase domain. The dashed line represents the linker loop between the ZBD and RPD and is suggested to constrain the RPD rotation range and influence the length of the synthesized primer.
