# Supplementary material for: Structural basis of the T4 bacteriophage primosome assembly and primer synthesis
Source: Nat Commun. 2023 Jul 20;14:4396. doi: 10.1038/s41467-023-40106-2 (PMC10359460; doi:10.1038/s41467-023-40106-2)
Supplement: Supplementary file 6 — Reporting Summary [file 41467_2023_40106_MOESM6_ESM.pdf]

nature portfolio

Corresponding author(s): Stephen Benkovic and Huilin Li

Last updated by author(s): Jun 25, 2023

Reporting Summary

Nature Portfolio wishes to improve the reproducibility of the work that we publish. This form provides structure for consistency and transparency in reporting. For further information on Nature Portfolio policies, see our Editorial Policies and the Editorial Policy Checklist.

Statistics

For all statistical analyses, confirm that the following items are present in the figure legend, table legend, main text, or Methods section.

n/a	Confirmed	
☐	☒	The exact sample size (*n*) for each experimental group/condition, given as a discrete number and unit of measurement
☐	☒	A statement on whether measurements were taken from distinct samples or whether the same sample was measured repeatedly
☒	☐	The statistical test(s) used AND whether they are one- or two-sided<br>*Only common tests should be described solely by name; describe more complex techniques in the Methods section.*
☒	☐	A description of all covariates tested
☒	☐	A description of any assumptions or corrections, such as tests of normality and adjustment for multiple comparisons
☒	☐	A full description of the statistical parameters including central tendency (e.g. means) or other basic estimates (e.g. regression coefficient) AND variation (e.g. standard deviation) or associated estimates of uncertainty (e.g. confidence intervals)
☒	☐	For null hypothesis testing, the test statistic (e.g. *F*, *t*, *r*) with confidence intervals, effect sizes, degrees of freedom and *P* value noted<br>*Give P values as exact values whenever suitable.*
☒	☐	For Bayesian analysis, information on the choice of priors and Markov chain Monte Carlo settings
☒	☐	For hierarchical and complex designs, identification of the appropriate level for tests and full reporting of outcomes
☒	☐	Estimates of effect sizes (e.g. Cohen's *d*, Pearson's *r*), indicating how they were calculated

Our web collection on statistics for biologists contains articles on many of the points above.

Software and code

Policy information about availability of computer code

Data collection	Cryo-EM data collection used SerialEM (version 3.8.8) in Titan Krios.
Data analysis	RELION-3, RELION-3.1, MotionCorr2-1.4.0, CTFFIND 4.1.10, cryoSPARC2 (v3.2), UCSF PyEM (version 0.5), ChimeraX 1.2.5, Coot (version 0.9.5), Phenix (version 1.20-4459-000), Pymol (version 1.8.x), AlphaFold (v2.1.1), MolProbity (version 4.5), DeepEMhancer (v0.14).

For manuscripts utilizing custom algorithms or software that are central to the research but not yet described in published literature, software must be made available to editors and reviewers. We strongly encourage code deposition in a community repository (e.g. GitHub). See the Nature Portfolio guidelines for submitting code & software for further information.

Data

Policy information about availability of data

All manuscripts must include a data availability statement. This statement should provide the following information, where applicable:

- Accession codes, unique identifiers, or web links for publicly available datasets
- A description of any restrictions on data availability
- For clinical datasets or third party data, please ensure that the statement adheres to our policy

The protein data bank accession codes for the atomic coordinates reported in this paper are 8DUO for the open spiral of the gp41 helicase hexamer; 8DTP for the closed ring of the gp41 helicase hexamer bound to ssDNA; 8DUE for the open spiral of the gp41 helicase hexamer bound to ssDNA; 8DVF, 8DVI, and 8DW6 for the T4 primosome in pose 1, pose 2, and pose 3, respectively; and 8G0Z, 8DWJ, and 8GAO for the helicase region, primase region and the whole primosome,

respectively, of a mutant T4 primosome bound to an RNA primer/DNA hybrid in a post RNA primer-synthesis state. The EM data bank accession codes for the 3D EM maps reported in this paper are EMD-27724 for the open spiral of the gp41 helicase hexamer (map 1-I); EMD-27720 and EMD-27719 for the open spiral of the gp41 helicase hexamer bound to ssDNA (map 2-I and map 3-I); EMD-27708 and EMD-27707 for the closed ring of the gp41 helicase hexamer bound to ssDNA (map 2-II and map 3-II helicase region); and EMD-27737, EMD-27739, and EMD-27751 for the WT T4 primosome bound to ssDNA in poses 1, 2, and 3, respectively. EMDB-27707, EMDB-29744, and EMD-29902 are deposited for the helicase region, primase region and a composite map of the mutant T4 primosome bound to an RNA primer/DNA hybrid, respectively. EMDB-29744 is a local refinement from map EMDB-27756, which is deposited as well.

Human research participants

Policy information about studies involving human research participants and Sex and Gender in Research.

Reporting on sex and gender	N/A
Population characteristics	N/A
Recruitment	N/A
Ethics oversight	N/A

Note that full information on the approval of the study protocol must also be provided in the manuscript.

Field-specific reporting

Please select the one below that is the best fit for your research. If you are not sure, read the appropriate sections before making your selection.

☒ Life sciences ☐ Behavioural & social sciences ☐ Ecological, evolutionary & environmental sciences

For a reference copy of the document with all sections, see nature.com/documents/nr-reporting-summary-flat.pdf

Life sciences study design

All studies must disclose on these points even when the disclosure is negative.

Sample size	No sample size calculation was performed. The gp41 helicase-DNA/RNA primer dataset contained 4091 movie stacks. The wild-type primosome with DNA/RNA primer dataset contained 11476 movie stacks. The dataset of the mutant primosome bound to ssDNA/RNA primer contained 10,224 movies. The data sizes were deemed sufficient because they had led to the refined EM maps at our target resolutions.
Data exclusions	"Bad" raw particle images of the helicase or primosome complexes that did not produce 2D class averages or 3D class maps with defined features were excluded after 2D and 3D classifications. This criteria is empirical but is a standard image processing practice in the cryoEM community.
Replication	The unwinding and priming assays were performed in triplicate. No replicate experiments were performed for cryo-EM experiments. Reproducibility resides in the large number of particles used to derive at the final 3D maps or 2D averages. The reliability and the resolution is measured by gold-standard Fourier shell correlation. Replication efforts with multiple refinement runs yielded was successful, yielding similar 3D maps.
Randomization	The raw particles were automatically selected by computer program (RELION-3.1). Randomization is not needed in cryo-EM analysis, because the particles were randomly distributed in the dataset.
Blinding	The investigators were not blinded to the specific data points during data collection and analysis, because visual inspection is necessary to ascertain the data quality.

Reporting for specific materials, systems and methods

We require information from authors about some types of materials, experimental systems and methods used in many studies. Here, indicate whether each material, system or method listed is relevant to your study. If you are not sure if a list item applies to your research, read the appropriate section before selecting a response.

Materials & experimental systems

n/a	Involved in the study
☒	☐ Antibodies
☒	☐ Eukaryotic cell lines
☒	☐ Palaeontology and archaeology
☒	☐ Animals and other organisms
☒	☐ Clinical data
☒	☐ Dual use research of concern

Methods

n/a	Involved in the study
☒	☐ ChIP-seq
☒	☐ Flow cytometry
☒	☐ MRI-based neuroimaging
